# Epidemiological characteristics of varicella from 2000 to 2008 and the impact of nationwide immunization in Taiwan

**DOI:** 10.1186/1471-2334-11-352

**Published:** 2011-12-16

**Authors:** Luan-Yin Chang, Li-Min Huang, I-Shou Chang, Fang-Yu Tsai

**Affiliations:** 1 Department of Pediatrics, National Taiwan University Hospital, College of Medicine, National Taiwan University, Taipei, Taiwan; 2 National Institute of Cancer Research and Division of Biostatistics and Bioinformatics, National Health Research Institute, Miaoli, Taiwan

**Keywords:** varicella, chickenpox, epidemiology, incidence, vaccine, prevention

## Abstract

**Background:**

Varicella has an important impact on public health. Starting in 2004 in Taiwan, nationwide free varicella vaccinations were given to 1-year-old children.

**Methods:**

Our study investigated the epidemiological characteristics of varicella from 2000 to 2008, and assessed the change of varicella epidemiology after the mass varicella immunization. ICD-9-CM codes related to varicella or chickenpox (052, 052.1, 052.2, 052.7, 052.8, 052.9) were analyzed for all young people under 20 years of age through the National Health Insurance database of Taiwan from 2000 to 2008.

**Results:**

Case numbers of varicella or chickenpox significantly declined after the nationwide immunization in 2004. Winter, particularly January, was the epidemic season of varicella. We found a significant post-vaccination decrease in incidence among preschool children, especially 3 to 6 year-old children-- the peak incidence was 66 per thousand for 4 and 5 year-old children before the nationwide immunization (2000 to 2003), and the peak incidence was 23 per thousand for 6 year-old children in 2008 (p < 0.001). Varicella-related hospitalization also significantly decreased in children younger than 6 years after the nationwide immunization.

**Conclusion:**

The varicella annual incidence and varicella-related hospitalization markedly declined in preschool children after nationwide varicella immunization in 2004.

## Background

Varicella is the primary disease caused by the varicella-zoster virus. It is a common and highly contagious disease and has a significant health impact on children. Although the clinical course of varicella is usually mild and self-limiting, varicella does cause complications and mortality resulting in financial expense [1-3]. Chickenpox or varicella now has been considered one of the most common vaccine-preventable diseases in many countries [4-7].

In Taiwan, some areas including Taipei City, Taichung City and Taichung County gave the public free varicella vaccinations for 1-2 year-old children before 2004. Taipei City started the free varicella vaccination from 1998 and Taichung City and Taichung County from 1999. Other areas did not provide free varicella vaccination, but people could receive varicella vaccination at their own expense before 2004. Since 2004, free mass varicella immunization has been given to all one year-old children throughout Taiwan.

Since the implementation of National Health Insurance (NHI) in 1995, there has been a healthcare database in Taiwan. Taiwan has a population of 22.9 million people and the land area of 36188 Km^2^. Its population density is 633/Km^2^. Taiwan's NHI covered most of the health care costs for 98% of its population in 2006 [8]; the remaining 2% of it population are living in the foreign countries or in families with monthly household incomes less than 1000 US dollars. Taiwan's NHI database includes health care data collected from over 95% of the hospitals in Taiwan for more than 96% of the population receiving health care. We evaluated the disease burden and epidemiological characteristics of varicella by using the NHI database to find out the age-specific incidences, seasonal distribution and varicella-related hospitalization from 2000 to 2008-- the period covering pre- and post-public free vaccination-- so we could compare the epidemiological characteristics of varicella before and after the mass immunization.

## Methods

### Vaccination

The administration of a live attenuated varicella vaccine was licensed in Taiwan in 1997. Both Merck and GlaxoSmithKline, pharmaceutical companies, provided the varicella vaccines in Taiwan. In 2004 the varicella vaccine was incorporated into Taiwan's national immunization program, and all 1-year-old children could receive the varicella vaccine for free. The varicella coverage rate for 1-year-old children was 94% for the 2003 birth cohort, 95% for the 2004 cohort, and 97% for the 2005, 2006, and 2007 birth cohorts according to Taiwan's National Immunization Information System which was developed in the early 1990s [9]. When a child received a vaccine, his or her vaccination would not only be recorded in a child's vaccination handbook but would also be reported to the National Immunization Information System.

### Data collection

Taiwan's National Health Insurance (NHI) covered most of the health care costs for 98% of its population. Taiwan's NHI database includes health care data collected from over 95% of the hospitals in Taiwan for more than 98% of the population receiving health care. From this database, hospitalization and outpatient healthcare records were collected from 2000 to 2008, and International Classifications of Diseases, Ninth Revision, Clinical Modification (ICD-9-CM) codes related to varicella or chickenpox were analyzed for all the population. The age-specific annual incidence and hospitalization rate were calculated. For calculating the annual population-based incidence, the annual incidence was calculated by dividing the number of varicella-related diseases by the population, which was obtained from Department of Statistics, Ministry of the Interior, Taiwan from 2000 to 2008 [10].

### Definitions

ICD-9-CM codes for chickenpox or varicella include the following: 052 chickenpox; 052.0 post-varicella encephalitis, post-chickenpox encephalitis; 052.1 varicella (hemorrhagic) pneumonitis; 052.7 with other specified complications; 052.8 with unspecified complication; 052.9 varicella without mention of complication.

For complications, we define varicella or chickenpox patients to have complications in cases where the patients had both ICD-9-CM codes for varicella or chickenpox (052 and the other related codes) and also ICD-9-CM codes for varicella-related complications which include the following: central nervous system including 320 meningitis, 322 cerebellitis, 323 encephalitis, 348 encephalopathy, 351 facial palsy, 331.81 Reye's syndrome, 780.3 febrile convulsion/seizure; skin and soft tissue including 680-686 cellulites and abscess, 035 erysipela, 728 pyomyositis and necrotizing fasciitis, 373 & 376.01 blepharitis, 034 & 041 scarlet fever and streptococcal or staphylococcal infection; skeletal system including 711 arthritis, 730-733 osteomyelitis; lower respiratory tract infection including 480-487 pneumonia, 510-519 pneumonitis, 466 & 490 bronchitis; hematological system- 287 thrombocytopenia, 283 & 285 anemia, 288 neutropenia; 038 & 790 & 995.91-995.92 for sepsis and bacteremia; 040-041 for other bacterial infection; 422 cardiomyopathy, 425 myocarditis; 070.5 & 070.9 & 573 for hepatitis.

### Statistics

In univariate analysis, categorical variables were compared with chi-square or Fisher's exact test; continuous variables were analyzed with Student's *t *test. The difference of annual incidence among various age groups, the difference of annual incidences in different years, and the difference in seasonal distribution were measured with appropriate χ^2 ^test. A *p *value of < 0.05 was considered to be statistically significant. All statistical analyses were performed with SAS software, version 9.0.

## Results

### Number of cases before and after the mass immunization

**Figure 1:**
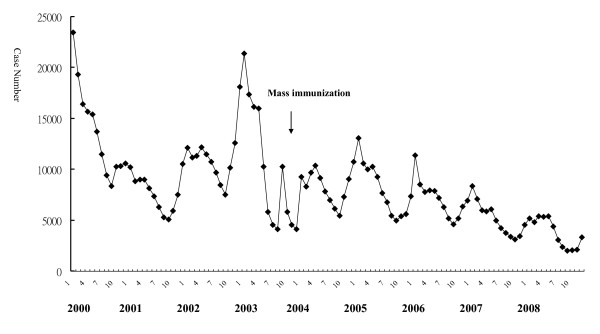
**Monthly distribution of varicella case number from 2000 to 2008**.

The monthly distribution of case numbers from 2000 to 2008 is demonstrated in Figure 1. Case numbers markedly declined after the mass immunization in 2004, the highest monthly number was 23460 in January 2000, and the lowest was 2016 in September 2008. The peak of varicella usually occurred during winter, especially in January.

### Age distribution

**Figure 2:**
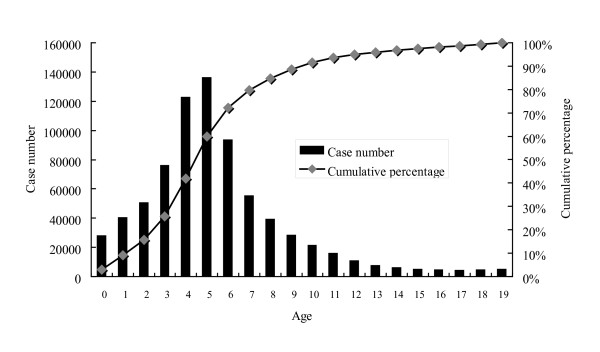
**Age-specific case number and cumulative percentage of varicella in children from 2000 to 2008**.

**Figure 3:**
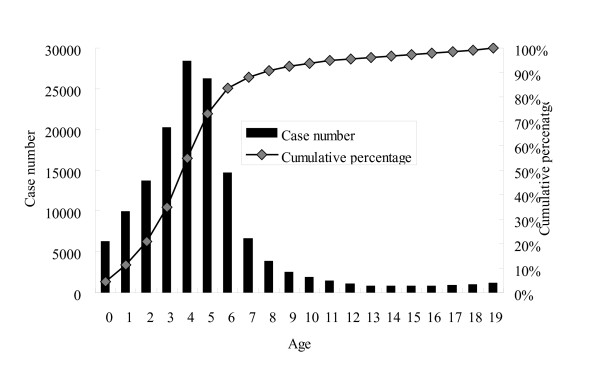
**Age-specific case number and cumulative percentage of varicella in children in 2000, before the nationwide immunization**.

**Figure 4:**
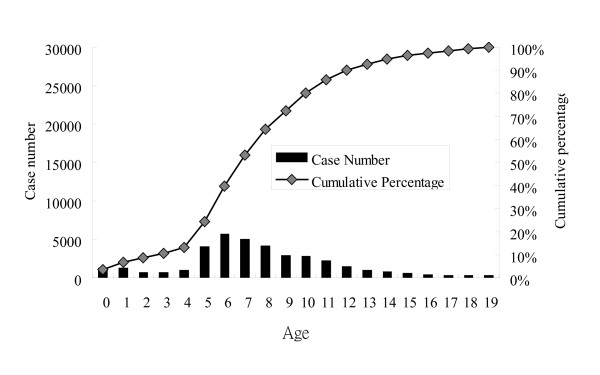
**Age-specific case number and cumulative percentage of varicella in children in 2008, 4 years after the nationwide immunization**.

Overall age-specific case numbers and the cumulative percentage of varicella in children from 2000 to 2008 are shown in Figure 2, those of 2000 in Figure 3, and those of 2008 in Figure 4. Children younger than 10 years old represented 89% of all the cases and the peak age was 3 to 6 years from 2000 to 2008 (Figure 2). However, the age distribution changed significantly after the mass immunization (p < 0.001): 73% of varicella cases were children less than 6 years of age in 2000 (before the mass immunization, Figure 3) but only 24% of the cases were children younger than 6 years of age in 2008 (4 years after the mass immunization, Figure 4). In comparison with age-specific case numbers of 2000 (Figure 3), those of 2008 were significantly lower (Figure 4) (p < 0.01). In 2008, school children had more varicella cases than the kindergarteners.

### Age-specific annual incidence

**Figure 5:**
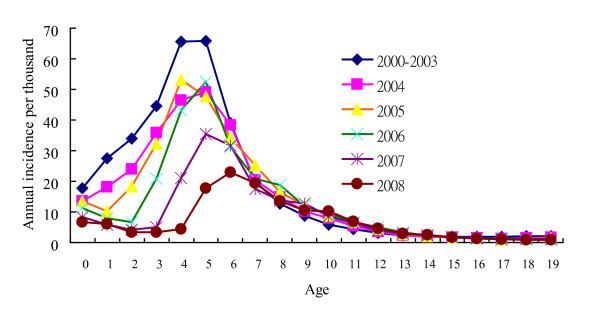
**Age-specific annual incidence of varicella before (2000-2003) and after the nationwide immunization (2004, 2005, 2006, 2007 and 2008)**.

Figure 5 demonstrates the age-specific annual incidence of varicella before and after the nationwide immunization. In 2000 to 2003 (before the nationwide immunization), the peak incidence was in 4 and 5-year-old children with annual incidence of 66 per thousand while in 2008, the peak incidence occurred in 6-year-old children with annual incidence of 23 per thousand (p < 0.01). After the nationwide immunization in 2004, the annual incidence significantly decreased among children younger than 7 years of age. In 2008, the annual incidence (6.8 per thousand) of infants who could not receive the varicella vaccine was even higher than that (3.2 to 5.9 per thousand) of 1 to 4-year-old children. Children beyond 7 years had similar annual incidence before and after the mass immunization.

### Age-specific varicella-related hospitalization incidence

**Figure 6:**
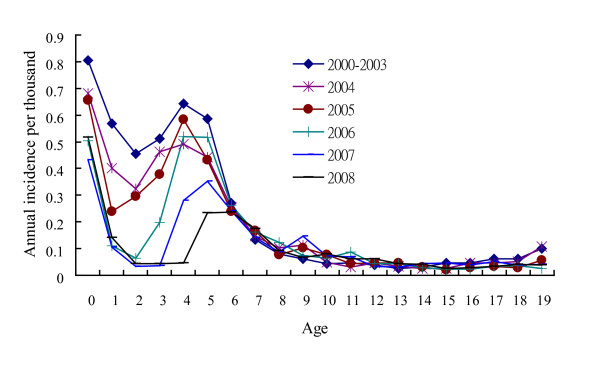
**Age-specific annual incidence of varicella-related hospitalization before (2000-2003) and after the nationwide immunization (2004, 2005, 2006, 2007 and 2008)**.

The proportion of the number of varicella-related hospitalization to the number of overall varicella cases was the highest in infants and declined with age in children. Figure 6 shows that the annual incidence of varicella-related hospitalization was the highest in infants. In comparison with the annual incidence of 2000 to 2003 (before the nationwide immunization), the annual incidence of hospitalization significantly declined in children younger than 6 years of age from 2004 to 2008 (p < 0.01). From 2000 to 2008, the annual incidence of preschool children decreased obviously with the year.

### Complications

Among the 21829 hospitalized cases, 561 (2.57%) had central nervous system complications, 2721 (12.47%) had skin or soft tissue infections, 4740 (21.71%) had lower respiratory tract infections, 493 (2.26%) had hematological complications, 281 (1.29%) had septicemia or bacteremia, and 721 (3.30%) had hepatitis.

Twelve children died and Table 1 lists the demography and complications of the 12 fatal varicella cases. Five of them had underlying diseases including 2 with acute lymphoblastic leukemia, 1 with severe combined immunodeficiency, 1 with enzymopathy, and 1 with pulmonary vascular anomaly. Most of them had complications of septicemia, skin or soft tissue infections, or encephalitis, and 1 had Reye syndrome.

**Table 1:** Demography and complications of 12 fatal varicella cases

Year	Age	Sex	Underlying disease	Complications
2000	1	F	Nil	Encephalitis, septic shock
2000	1	M	Unspecified Enzymopathy	Pseudomonas septicemia
2000	4	F	Acute lymphoblastic leukemia	Septicemia
2001	5	F	Nil	Encephalitis, septicemia
2001	6	F	Nil	Sudden death
2002	3	F	Nil	Respiratory failure
2002	3	M	Acute lymphoblastic leukemia	Not mentioned
2003	2	F	Nil	Skin/soft tissue infections, streptococcal septicemia
2004	16	M	Nil	Skin/soft tissue infections, pneumonia, septicemia
2005	1	F	Nil	Reye's syndrome
2006	1	M	Severe combined immunodeficiency	Acute and subacute hepatic necrosis, gastrointestinal hemorrhage
2007	0	M	Pulmonary vascular anomaly	Hemoptysis, acute respiratory failure

## Discussion

Nationwide varicella immunization has resulted in a marked reduction of varicella incidence and varicella-related hospitalization in children younger than 6 years of age in Taiwan. The impact of mass varicella immunization has so far made it possible for most preschool children to be free of varicella and varicella-related hospitalization in Taiwan. However, children beyond 7 years of age had similar annual incidence before and after the mass immunization.

As Figure 3 shows the age distribution before the mass immunization in 2000, the peak age of varicella was 3 to 6 years, which was also the age group of kindergarteners. Kindergarteners might have more exposure to varicella and also their hand hygiene and sanitation levels are generally not as high as adults' or older school children's, who may in turn lead to more varicella cases in kindergarteners without mass immunization (Figure 3). However, after the mass immunization, we observed more varicella cases in the school children than those in 3 to 4-year-old kindergarteners (Figure 4), who were supposed to have received the free varicella vaccine at 1 year of age. Therefore, mass immunization has changed the age distribution of varicella cases significantly and the peak age has subsequently moved to older children without previous immunization. Similar results were also reported by Lian et al who found that the greatest decrease in varicella incidence occurred in children aged below 6 and the incidence of varicella shifted to older age groups after implementation of the one-dose varicella vaccination policy [11]. Lian et al also found that the incidence in early launch areas was significantly lower than that in the other areas in Taiwan [11]. Our study additionally reported the seasonality, varicella-related hospitalization and complications from 2000 to 2008 in Taiwan. Moreover, our data in this study is nationwide rather than the selected sample size like that of Lian et al's study.

We found that the annual incidence of children beyond 7 years of age was similar before and after mass immunization. It's because most of the school children were not allowed to receive the free varicella vaccine. Thus, the varicella incidence of the school children did not change significantly after the mass immunization in this study. We suppose that the annual incidence of school children may decrease several years later if the immunized children grow up to be 6 to 12 years old. However, we have to follow up on the impact of mass immunization on school children to prove the above assumption several years later.

The infants had the highest annual incidence of varicella-related hospitalization both before and after the mass immunization (Figure 6). Infants cannot receive the varicella vaccine and they have a higher chance of severe varicella and more complications [12], so their varicella-related hospitalization was significantly higher than the other children.

There were 12 cases of fatality, of which 5 of them had underlying diseases. Children with underlying diseases such as acute leukemia are more susceptible to getting severe varicella with complications and have a significantly higher mortality rate [12]. Therefore, the varicella vaccine may be administered to children with acute lymphoblastic leukemia who are in remission, or, high-titer anti-varicella-zoster virus immune globulin as post-exposure prophylaxis is recommended for immunocompromised children and newborns that are exposed maternally to varicella [12].

Currently, the varicella vaccine program consists of a single-dose vaccination in Taiwan, as it is in Canada, Korea and Australia [13]. However, both varicella outbreaks and breakthrough infections have occurred after the single-dose varicella vaccine program was initiated [14]. These might be related to a loss of immunity or to primary vaccine failure. Although the reports are limited, varicella vaccine effectiveness may be higher when two doses are administered than when a single-dose is administered. In 2006, the United States changed its immunization policy by stipulating that 2 doses be administered, once at 1 year of age and another administered between the ages of 3 and 4 years old [15]. In Taiwan, boosters in 4-6 year-old children to reduce the breakthrough infection or amend the primary vaccine failure may be considered if further epidemiology reveals frequent outbreaks or breakthrough infections in kindergarteners.

The major limitation of this study is that we could not verify the diagnoses because we could just analyze the NHI database rather than detailed medical charts. However, at the very least the nationwide database can let us outline the comprehensive picture of varicella epidemiology in Taiwan and sketch the impact of nationwide immunization.

## Conclusions

In conclusion, nationwide varicella immunization significantly decreased the varicella annual incidence and varicella-related hospitalization in preschool children in Taiwan. The impact of the varicella vaccine on schoolchildren may be followed up on several years later.

## Competing interests

The authors declare that they have no competing interests.

## Authors' contributions

LYC, LMH and ISC designed the study. LYC and FYT analyzed the data. LYC wrote the paper. All authors read and approved the final manuscript.

## Pre-publication history

The pre-publication history for this paper can be accessed here:

http://www.biomedcentral.com/1471-2334/11/352/prepub
